# Climate-Driven Variability in the Chemical Composition and Antioxidant Activity of *Allium ursinum* L.

**DOI:** 10.3390/antiox14121477

**Published:** 2025-12-09

**Authors:** Jolita Radušienė, Birutė Karpavičienė, Kristina Zymone, Mindaugas Marksa, Lina Raudone

**Affiliations:** 1 Laboratory of Economic Botany, Nature Research Centre, Akademijos Street 2, 08412 Vilnius, Lithuania; jolita.radusiene@gamtc.lt (J.R.); birute.karpaviciene@gamtc.lt (B.K.); 2 Department of Analytical and Toxicological Chemistry, Lithuanian University of Health Sciences, Sukileliu Av. 13, 50162 Kaunas, Lithuania; kristina.zymone@lsmu.lt (K.Z.); mindaugas.marksa@lsmu.lt (M.M.); 3 Laboratory of Biopharmaceutical Research, Institute of Pharmaceutical Technologies, Lithuanian University of Health Sciences, Sukileliu Av. 13, 50162 Kaunas, Lithuania; 4 Department of Pharmacognosy, Lithuanian University of Health Sciences, Sukileliu Av. 13, 50162 Kaunas, Lithuania

**Keywords:** phenolic compounds, antioxidant activity, flavonol glycosides, sulfur compounds, *Allium ursinum*, allicin, lutein

## Abstract

*Allium ursinum* L. (wild garlic) is a valuable medicinal and culinary plant, rich in bioactive compounds with antioxidant properties. This study evaluated the chemical composition and antioxidant activity of *A. ursinum* populations growing across eleven distinct sites in Lithuania, representing their different habitats. Leaves and flowers were extracted using solvent systems optimized for different compound groups, 70% methanol for phenolics, purified water for sulfur compounds, and methanol for carotenoids, assisted by ultrasonic extraction. Using HPLC-PDA and spectrophotometric assays, major flavonol glycosides, sulfur compounds and carotenoids were quantified in leaves and flowers. Significant variability in compound concentrations was observed between populations and plant organs. Flowers accumulated allicin (622–1442 μg/g DM) and higher levels of flavonol derivatives (up to 5949 μg/g DM), whereas leaves contained more carotenoids (384–656 μg/g DM). Antioxidant activity ranged from 473 to 719 μmol TE/g DM and showed positive correlation with the total content of identified phenolics in flowers. However, no significant correlation was observed between total phenolics and total antioxidant capacity in leaves. Multivariate analysis revealed clear clustering of populations based on climatic parameters, with higher precipitation and moderate spring temperatures favoring higher phenolic content. These findings suggest that local environmental factors significantly influence phytochemical profiles and antioxidant potential in *A. ursinum*. The results emphasize the importance of habitat conditions for optimal yield of bioactive compounds and support the development of site-adapted cultivation strategies for high-quality production of *A. ursinum* raw material.

## 1. Introduction

*Allium ursinum* L., commonly known as wild garlic or ramsons, is a perennial herbaceous species distributed across European temperate forests, from the Mediterranean to Scandinavia, except in northern areas, the evergreen Mediterranean region, and high altitudes [1]. *A. ursinum* grows in moist but well-drained deciduous forests, riverbanks, and mountain valleys, from lowlands up to 1800 m.a.s.l. (above sea level) preferring humus-rich, slightly acidic to neutral soils [2]. *Allium ursinum* is an early spring perennial geophyte that each year produces two foliage leaves and a new bulb about 1.5 to 6 cm in size, which develops from the base of the upper leaf. *A. ursinum* usually reproduces by seed, with limited vegetative propagation being useful under adverse environmental conditions [3,4]. Due to intensive reproduction, *A. ursinum* forms abundant and dense monospecific stands with up to 3350 individuals per m^2^ [5]. The main growth and reproduction of *A. ursinum* occur over a very short period, from the onset of favorable growth conditions in early spring to the closure of the overhead canopy, thus avoiding interspecific competition for light [6,7].

The narrow ecological tolerance of *A. ursinum* poses a challenge for cultivation, as it requires specific environmental conditions that are difficult to reproduce [8]. Researchers explored unconventional agrotechnological treatments for cultivating *A. ursinum* in field conditions, intercropping companion plants such as phacelia and rapeseed, or horseradish to create artificial shade [9,10]. *A. ursinum* is also difficult to maintain using micropropagation because of its specific soil requirements, slow growth, and poor germination rate [11].

Thus, the main raw material for wild garlic comes from wild populations, which may pose a risk of excessive exploitation. Overharvesting and habitat loss due to urban development or logging pose threats at the edge of *A. ursinum* distribution range [12]. Although *A. ursinum* is not considered threatened at the European level, wild garlic is included in the list of protected species in several countries, including Latvia [13], Estonia [14], Ukraine [15], and Belarus [16]. Meanwhile, *A. ursinum* was included in the Lithuanian Red Book in 1962, but in 2019, it was reclassified as near-threatened and is no longer protected. However, after assessing information that people began collecting wild garlic leaves in large quantities and selling them as a spring herb, this species was included in the list of restricted-use wild plant and mushroom species in 2023 [17].

All parts of *A. ursinum* are edible and have culinary uses, and the plant material can be classified as a functional food [18]. Traditionally, young fresh leaves of *A. ursinum* are consumed as a spring vegetable, and dried leaves are used as a spice [19]. Since ancient times, *Allium* plants have been important components of typical recipes and traditional treatment systems [20]. In European traditional medicine, ramson is generally recommended as a digestive stimulant, an antimicrobial agent, a detoxifier, and a preventive measure against cardiovascular disease [4]. It is effective in reducing high blood pressure and cholesterol levels [21,22]. Its alcoholic extract inhibits platelet aggregation, thus protecting against coronary heart disease [23]. *A. ursinum* possesses strong antioxidant activity, which implicates its use in the prevention of cardiovascular diseases or as an adjuvant to antioxidant therapy [24]. Its extracts exhibit fungistatic [25], antibacterial [26,27,28], antiparasitic [29], and antithyroinase activity [30].

*Allium ursinum* is recognized for its exceptional taste and rich phytochemical composition. The key biologically active substances in *A. ursinum* responsible for the effects on maintaining good health are sulfur-containing compounds (allicin, ajoene), which play a crucial role in determining the characteristic odor and taste of the plant [4,31]. In the intact cell, the sulfoxides as flavor precursors are compartmentalized in the cytoplasm, and the hydrolytic enzyme alliinase is in the vacuole. When plant tissue is damaged, alliin is hydrolyzed by the enzyme alliinase and converted to allicin [32,33]. Organic sulfur compounds predominate in the essential oil of *A. ursinum*, with a relative content up to 98% [26,34]. Besides sulfur compounds, all parts of the plant contain a wide range of secondary metabolites, e.g., phenolic compounds, triterpenoids, carotenoids, and steroidal glycosides [35,36,37,38,39]. Flavonoids, particularly flavonols from alliums, contribute to their antioxidant, antimicrobial, anti-inflammatory, and cardioprotective properties [40,41].

While many studies have documented the bioactive composition of *A. ursinum,* most have focused on single locations or cultivated material, often neglecting the potential role of environmental factors in modulating phytochemical variability [26,42]. Given its widespread distribution across diverse habitats, *A. ursinum* serves as an excellent model for studying environmentally driven plasticity in the secondary metabolism of forest herbs. In Lithuania, the natural variability in the phytochemistry of *A. ursinum* has not been systematically investigated. Understanding this variability is crucial for both conservation and optimized commercial cultivation, as environmental conditions can significantly impact the yield of desirable bioactive compounds. This study is the first to combine targeted quantification of phenolic, sulfur, and carotenoid compounds with site-specific climatic data across multiple natural populations of *Allium ursinum* in Lithuania. By linking chemical composition to local temperature and precipitation patterns, our results provide novel insights into the environmental modulation of secondary metabolism at the northeastern distribution margin of the species.

The primary objectives of this study were: (1) to identify and quantify major phenolic, flavonoid, carotenoid, and sulfur compounds in leaves and flowers of *A. ursinum*; (2) to evaluate antioxidant activity of the plant extracts and correlate it with phytochemical composition; (3) to analyze relationships between environmental (climatic) parameters and the chemical profiles of different populations and provide recommendations for conservation and sustainable cultivation based on identified phytochemical–environment interactions.

The obtained results will complement the knowledge of *A. urssinum* species diversity across different European regions, assessing environmental conditions for cultivation potential in the face of increasing threats to its natural habitats.

## 2. Materials and Methods

### 2.1. Sampling Sites

**Figure 1:**
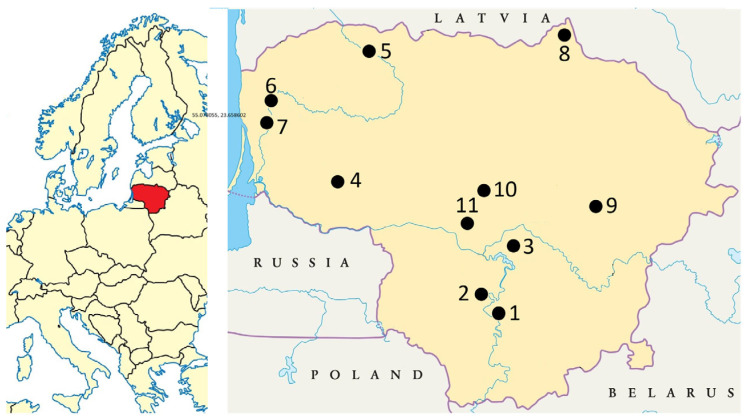
Sampling sites of *Allium ursinum* in Lithuania, 2024.

Lithuania lies on the East European Plain (54–56° N, 21–25° E) within a temperate, hemiboreal climate zone characterized by mild summers and cold winters. The country is mostly flat, with the highest point reaching 297.8 m.a.s.l. The long-term average temperature was 7.4 °C, and the average annual precipitation was 695 mm, with most of it falling in summer. The growing season lasts 169 days in the east and 202 days in the western part of Lithuania [43]. *Allium ursinum* raw material was collected from 11 populations located between 54.511944–56.34343° N and 21.558562–25.343055° E, at an elevation of 56–186 m.a.s.l., from 8 to 17 May 2024. Five populations were located in the central (sites 1, 2, 3, 10, 11), four in the western (sites 4, 5, 6, 7), and one each in the northern (site 8) and eastern (site 9) parts of the country (Figure 1).

**Figure 2:**
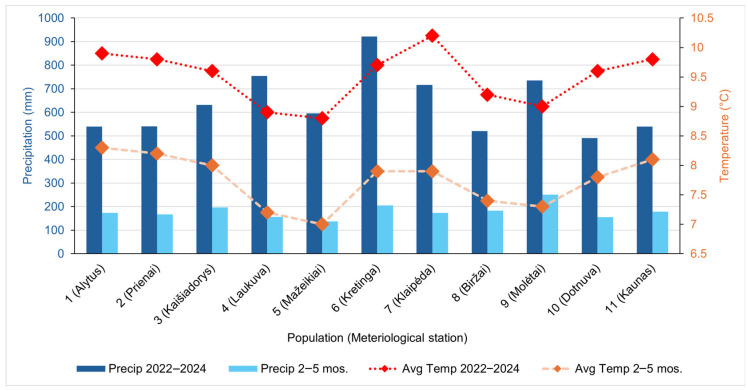
Climatic conditions, average annual and spring (February—May) air temperature (Avg Temp), and precipitation (Precip) for 20222024 at *Allium ursinum* sampling sites according to the data of the closest meteorological stations obtained from the Archive of Meteorological and Hydrological Observations [44].

The elevation across 11 *A. ursinum* sampling sites ranged only slightly and exerted little influence on microclimate. The average annual air temperatures at nearby meteorological stations of the sampling sites in 2022–2024 ranged from 8.8 °C (site 5) to 10.2 °C (site 7), while the average spring (February–May) temperatures ranged between 7.0 °C (site 5) and 8.3 °C (site 1). Total annual precipitation spanned 490.8 mm (site 10) to 921.4 mm (site 6), and spring rainfall from 155.7 mm (site 10) to 250.6 mm (site 9) (Figure 2).

The habitats of *A. ursinum* were mesic deciduous or mixed coniferous–deciduous forests on slightly acidic to neutral, humus-rich soils [45] in moist, spring-fed areas or on slopes along streams with a southern, western, or northern aspect, typically flooded in spring but well-drained later in the growing season. Population 2, on the northern slope of a spring-fed stream, stood out from all the others because the plants formed did not form inflorescences.

*Allium ursinum* formed dense monodominant stands covering several hectares in the mature and mid-mature mixed forests with rich undergrowth. The understory composition ranged from *Alnus glutinosa*–*Picea abies* stands in wetter lowlands (populations 1 and 6) to *Ulmus glabra–Quercus robur–Betula pendula* assemblages on drier slopes (populations 3, 4, and 10). Understory richness (e.g., *Corylus avellana*, *Ribes nigrum*, *Padus avium*, *Lonicera xylosteum*, *Daphne mesereum*) was high in all sites, consistent with the species’ preference for shaded, nutrient-rich habitats (Table 1).

**Figure 3:**
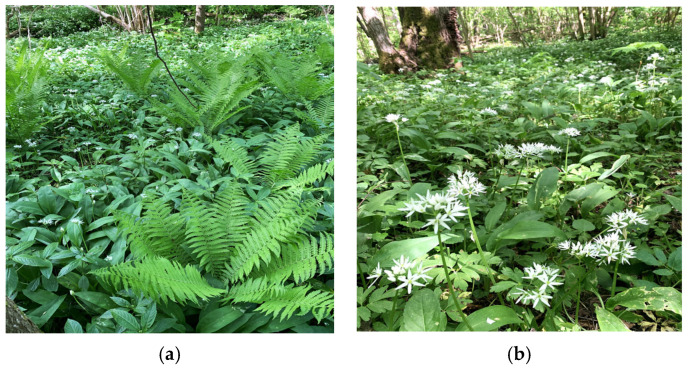
*Allium ursinum* 8 (**a**) and 9 (**b**) populations in mixed forest. Photo by authors.

*Allium ursinum* is a strong interspecific competitor, which affects the growth of other herbaceous plants. In the vegetation cover with the dominant *A. ursinum*, the most abundant herbal species were *Anemone ranunculoides* L., *Anemone nemorosa* L., *Geum rivale* L., *Aegopodium podagraria* L., *Dryopteris filix-mas* (L.) *Schott*, *Rubus idaeus* L., *Impatiens noli-tangere* L., *Stachys sylvatica* L., *Paris quadrifolia* L., and *Pulmonaria obscura* Dum. (Figure 3).

### 2.2. Plant Material

Wild garlic leaves appear in late February to March, depending on local conditions, and the plants begin to bloom in late April, finishing in late May. The plant material consisted of 30 healthy, undamaged plants per population (approximately 400–600 g of fresh material), randomly collected along the habitat transect during the flowering phase between 8 and 17 May 2024. The plant material from population 2 was included in the study because it reflects habitat differences. Plants were collected in the first half of the day during dry weather, placed in paper bags, and transported to the laboratory in cooled containers. Plants from one population were pooled to obtain one homogeneous sample representing the population, and then were dissected into inflorescences with peduncles and leaves with petioles. The plant material samples were dried in a drying cabinet (SSO-80 Isoterma, Wroclaw, Poland) at 25 °C temperature and 10% relative humidity for 24 h. The air-dried plant material samples were ground to a homogeneous powder using a Retsch 200 mill (Haan, Germany) and kept in the dark in sealed containers until extraction. Before extraction, three leaf and flower samples from each population were weighed in triplicate for further chemical analysis.

### 2.3. Chemicals and Solvents

All the materials and reagents used in this study were of analytical and chromatographic grade: 99.8% trifluoroacetic acid, acetonitrile, methanol, ethyl acetate, trimethylamine, sodium hydrogen phosphate dihydrate, sodium 1-heptansulphonate monohydrate, sodium dihydrogen phosphate dihydrate, sodium 1-heptanesulfonate quercetin-3-glucuronide (≥95.0%), astragalin (≥97.0%), p-coumaric acid (≥98%), lutein (≥90.0%), allicin (≥90%), and kaempferol (≥97.0%) were purchased from Sigma-Aldrich GmbH (Steinheim, Germany). The ABTS (2,2′-azino-bis(3-ethylbenzothiazoline-6-sulfonic acid)) and Trolox ((+)-6-hydroxy-2,5,7,8-tetramethylchroman-2-carboxylic acid) were purchased from Sigma–Aldrich (Buchs, Switzerland). Ethanol (96.3%) was purchased from Vilniaus degtinė, Vilnius, Lithuania. The purified deionized water (18.2 mΩ/cm) was obtained using a Millipore (Burlington, MA, USA) water purification system.

### 2.4. Extraction

For the extraction of phenolic compounds approximately 0.1 g (precise weight) of the dried plant material and 10 mL of 70% (*v*/*v*) methanol were subjected for 30 min in an ultrasonic bath Elmasonic P (Singen, Germany), subsequently filtered through a 0.2 µm pore size membrane filters (Carl Roth GmbH, Karlsruhe, Germany) and kept at 4 °C until analysis. The sulfur compounds (allicin) were extracted from the dried plant material samples of about 1.0 g (precise weight) with 10 mL of purified water in an ultrasonic bath Elmasonic P (Singen, Germany) for 10 min, then filtered through 0.2 µm pore size membrane filters (Carl Roth GmbH, Karlsruhe, Germany) and kept at 4 °C until analysis. For the analysis of the carotenoid lutein, the dried plant material samples of about 0.5 g (precise weight) were extracted in 10 mL of 100% *v*/*v* methanol for 30 min in an ultrasonic bath Elmasonic P (Singen, Germany), then filtered through a 0.2 µm pore size membrane filters (Carl Roth GmbH, Karlsruhe, Germany) and kept at 4 °C until analysis. All extractions were carried out in an ultrasonic water bath (Bandelin Sonorex Digital 10 P, Berlin, Germany), operating at 35 kHz and 240 W, at room temperature (22 ± 2 °C), without pH adjustment.

### 2.5. HPLC Analysis

Qualitative and quantitative analyses of lutein were performed using a Shimadzu Nexera X2 LC-30AD HPLC system (Shimadzu, Tokyo, Japan) equipped with an SPA-M20A diode array detector and an LCMS-2020 mass spectrometer (Shimadzu, Tokyo, Japan) using an ACE column (C18, 250 mm × 4.6 mm, 5 µm). The mobile phase consisted ethyl acetate (eluent A) and 0.05% trimethylamine in acetonitrile (eluent B) according to a gradient of 0–1 min—85% B, 1–10 min—85–95% B, 10–12 min—95–100% B, 12–28 min—100% B, 28–29 min—100–90% B, 29–38 min—90% B, and 38–39 min—90–85% B. Eluent flow rate was 0.6 mL/min, and injection volume 20 µL. The column was temperature-controlled, maintained at 25 °C. Chromatographic peak identification was performed based on the retention times of analytes and reference compounds, as well as by comparing their UV absorption spectra with those of the reference compounds The analysis of phenolic compounds was performed using a Waters 2695 Alliance system (Waters, Milford, MA, USA) with a Waters 2998 photodiode array detector (Waters, Milford, MA, USA), and the HPLC-MS system comprised a Shimadzu Nexera X2 LC-30AD HPLC system (Shimadzu, Tokyo, Japan) equipped with an LCMS-2020 mass spectrometer (Shimadzu, Tokyo, Japan). Phenolic origin compounds were analyzed using an ACE (ACT, Aberdeen, UK) column (C18, 150 mm 4.6 mm, particle size 3 μm). The mobile phase consisted of 0.05% trifluoroacetic acid (eluent A) and acetonitrile (eluent B) at a gradient mode of 0–30 min—15–30% B, 30–50 min—30–60% B, 50–55 min—60–90% B, 55–56 min—90% B, and 57 min—15% B. The flow rate was 0.5 mL/min, and the injection volume was 10 µL. The column was temperature-controlled, maintained at 15 °C. The LCMS-2020 mass spectrometer ESI conditions were selected as 350 °C for the interface temperature, 250 °C for the DL temperature, 400 °C for the heat block temperature, 1.5 L/min for the nebulising gas flow, and 10 L/min for the drying gas flow. The m/z range was 50–2000, with 0.1 m/z steps. Chromatographic peak identification was performed by comparing the retention times of analytes and reference compounds, as well as their UV absorption spectra with those of the reference compounds, using a diode-array detector. Additionally, their mass spectra were compared with published literature data and fragmentation patterns available in open-access databases (Table 2).

Allicin was quantified using a Waters 2695 Alliance system (Waters, Milford, MA, USA) equipped with a Waters 2998 photodiode-array detector. Separation was performed on a C18 column (YMC-Triart 150 mm × 3.0 mm). Gradient elution was performed using eluent A consisting of 10 mM sodium dihydrogen phosphate dihydrate and 5 mM sodium 1-heptanesulfonate (pH adjusted to 2.1 with phosphoric acid), and eluent B consisting of acetonitrile mixed with 10 mM sodium dihydrogen phosphate dihydrate and 5 mM sodium 1-heptanesulfonate (50:50, *v*/*v*; pH 2.1). The gradient program was as follows: 0 min—100% A, 5 min—70% A, 25 min—46% A, 26 min—0% A, and 30 min—return to 100% A. The flow rate was 0.4 mL/min, the injection volume was 10 µL, and the column temperature was maintained at 38 °C. Detection was performed at 208 nm. Allicin was identified and quantified by comparing retention times and UV absorption spectra with those of a reference standard.

All chemical analyses were performed in triplicate.

### 2.6. Quantification

Quantitative analysis was performed based on the dependence of the analyte peak area on analyte concentration in the test solution. Calibration curves were compiled using standard solutions. Quantification of lutein was performed using a lutein calibration curve, quantification of allicin was performed using (±)-L-aliin calibration curve, quantification of quercetin-3-glucuronide was performed using quercetin-3-glucuronide acid calibration curve, quantification of p-coumaric acid derivatives was performed using p-coumaric acid calibration curve, and quantification of other flavonols was performed using astragalin calibration curve. Contents of lutein were calculated at 420 nm, p-coumaric acid derivatives were calculated at a wavelength of 310 nm, contents of other flavonols at 350 nm, and contents of allicin at 208 nm. The results were adjusted to reflect values for absolutely dry mass (DM).

### 2.7. Antioxidant Activity Assay

The antioxidant activity was analyzed using the spectrophotometric (Genesys-10 UV/Vis spectrophotometer (Thermo Spectronic, Rochester, NY, USA) ABTS ((2,2′-azino-bis(3-ethylbenzothiazoline-6-sulfonic acid)) assay as described by Re et al. [48] and modified by Raudone et al. [49]. The test solution was prepared by mixing 3 mL of ABTS working solution with 20 µL of each sample extract. The absorbance change of the mixture was measured with a spectrophotometer at 734 nm. The radical scavenging activity results were expressed as antioxidant Trolox equivalents (TE) per gram of dry mass of plant material (µmol TE/g, DM).

### 2.8. Data Analysis

Results for each population were expressed as the mean ± standard deviation (SD) of three samples for leaves and flowers, and their triplicate analyses were performed (3 × 3). For nonparametric statistics, Kruskal–Wallis ANOVA was used to determine significant differences in chemical compounds among populations at a confidence level of *p* < 0.05. Variable sets between plant organs were tested using a *t*-test for dependent variables. Principal component analysis (PCA) was used to identify similarities and differences among the analyzed populations based on statistically independent variables. The datasets of chemical compounds in leaves and flowers were pooled together and used in PCA to obtain more compelling results for visualization. PCA was performed, accounting for factors with eigenvalues greater than 1. It was based on standardized variables. Pearson’s correlation analysis was performed to explain the relationships between variables and principal components, and the *p*-value obtained by testing the hypothesis in the nonlinear regression. The data were processed in Microsoft Excel 365 Version 2403 (Microsoft, Redmond, WA, USA) and analyzed using multivariate statistical analysis in Statistica 10.0 (StatSoft Inc., Uppsala, Sweden).

## 3. Results

### 3.1. Chemical Composition and Organ-Specific Differences

A total of 20 specialized metabolites were identified and quantified in the extracts of native *Allium ursinum* leaves and/or flowers. These included 18 flavonols and their derivatives, a coumaric acid derivative, the xanthophyll lutein, and the sulfur-containing compound allicin (Table 3). The total content of identified phenolic compounds (the sum of individual quantified compounds determined by HPLC) was significantly different (*P*_1_ < 0.001) in the populations tested and ranged from 3852 up to 12,959 and from 4693 up to 14,704 µg/g in the leaf and flower samples, respectively.

The chemical profiles of *A. ursinum* varied between plant parts, both qualitatively and quantitatively. Sixteen individual compounds were found in the leaves, and ten in the flowers. The average total content of all detected compounds was significantly higher in the flowers (9365 ± 2535 µg/g DM) than in the leaves (7787 ± 2089 µg/g DM). However, the total phenolic compound content did not differ significantly between plant organs (*p* > 0.05), suggesting that the organ-specific chemical profiles of *A. ursinum* are determined more by differences in the composition of individual compounds than by total phenolic concentration.

Allicin (compound 1), a major bioactive sulfur compound, was detected exclusively in flowers, with a mean content of 1085.0 ± 330.3 μg/g DM (Table 3). Its absence in leaves aligns with previous findings, which show that extraction methods and tissue types significantly influence allicin detection [28]. Meanwhile, four compounds (**2**, **3**, **4**, and **12**) were present in both leaves and flowers, though their concentrations differed. Lutein (compound **2**) was significantly more abundant in leaves than flowers (535.0 ± 110.3 vs. 32.6 ± 8.3 µg/g DM), consistent with studies showing higher carotenoid levels in vegetative organs [38]. Compound **4**, a kaempferol-hexosyl-acetyl-deoxyhexose-hexoside derivative, was the most abundant flavonol in leaves (2421.3 ± 1065.5 µg/g DM) and one of the dominant in flowers (2303.1 ± 993.5 µg/g DM), showing no significant difference between plant parts but strong population-dependent variation. Compound **3** was particularly abundant in flowers, with a mean value of 3446.8 ± 1236.6 µg/g DM, and one among the major compounds in leaves (mean 910.7 ± 633.3 µg/g DM). Similarly, compound **12** accumulated in both parts of the plant but more prominently in flowers (1595.0 ± 797.9 µg/g DM). Several kaempferol derivatives (compounds **7**, **11**, **13**, **17**, and **19**) were detected only in leaves, with the highest amounts found in populations 8, 5, 7, 3, and 6 (788.8 ± 221.2, 736.7 ± 36.8, 1012.0 ± 413.2, 1439.3 ± 228.6, and 750.6 ± 82.2 μg/g DM, respectively). Compounds **6**, **8**, **9**, **14**, and **16** were detected in leaves at the lowest concentrations among the detected phenolics in *A. ursinum*. Furthermore, compounds **5**, **15**, **18**, and **20** were detected exclusively in flowers, in moderate and small amounts.

### 3.2. Differences Across Populations

**Figure 4:**
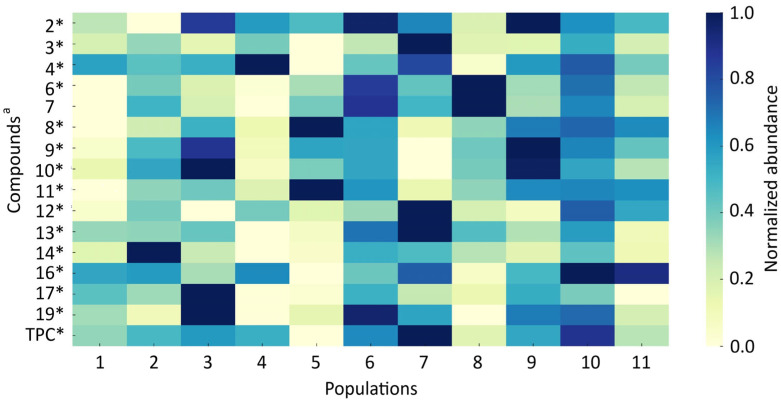
The heatmap of the relative abundance of the mean contents of individual compounds in *Allium ursinum* leaves across 11 populations. The background color of each cell represents the normalized mean value relative to the maximum for each compound. ^a^—the Compound numbers correspond to the numbering of the compounds listed in Table 3 *—significant differences among populations at *p* < 0.05 according to Kruskal–Wallis ANOVA; TPC—the sum of individual quantified phenolic compounds.

**Figure 5:**
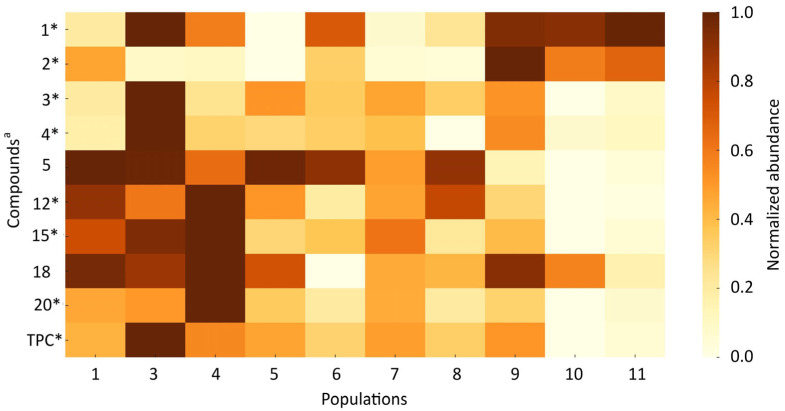
The heatmap of the relative abundance of individual compounds in *Allium ursinum* flowers across 11 populations. The background color of each cell represents the normalized mean values relative to the maximum value for each compound. ^a^—the compound numbers correspond to the numbering of compounds listed in Table 3. *—significant differences among populations at *p* < 0.05 according to Kruskal–Wallis ANOVA; TPC—the sum of individual quantified phenolic compounds.

The heatmaps present the normalized abundance profiles of the analyzed chemical compounds across 11 *A. ursinum* populations in leaves (Figure 4) and flowers (Figure 5).

The leaf populations **3**, **4**, **7**, and **10** consistently showed the highest overall abundances of several dominant compounds, indicating strong chemotypic clustering (Figure 4). Compounds **3**, **4**, and **13** were particularly elevated in corresponding populations, with population 4 exhibiting the highest concentration of compound **4** (4045.0 ± 687.3 µg/g DM). In contrast, compounds **3** and **13** peaked in population 7 (2550.5 ± 482.7 and 1012 ± 413.2 µg/g DM, respectively). Lutein (compound **2**) was relatively stable across populations, yet populations 6 and 9 displayed the highest mean levels (647.2 ± 82.9 and 655.7, respectively). Population 2 had the highest amount of compound **14** (quercetin glucuronide) (358.4 ± 42.3 µg/g DM).

A similarly distinct population-specific pattern was evident in the flowers (Figure 5). Populations 1, 3, 4, and 9 showed the greatest abundance of several specialized metabolites, particularly compounds **1**, **3**, **4**, and **12**. Allicin (compound **1**) displayed pronounced variability, with the highest concentrations found in populations 3, 9, 10, and 11 (up to 1442.9 ± 184.0 µg/g DM). Population 3 stood out for exceptionally high levels of compound **3** (5949.0 ± 1580 µg/g DM), consistent with the elevated total phenolic content observed in this population’s flowers. Compound **12** was most abundant in populations 1 and 4 (2467.9 ± 419.6 and 2704.7 ± 1029.5 µg/g DM, respectively). Notably, population 4 ranked among the top producers of compound 4 in both organs, confirming a strong population-specific chemical signature.

The sum of individual phenolics also varied significantly between populations. In leaves, phenolic levels ranged from 3852 to 12,959 µg/g DM, with populations 7 and 10 exhibiting the highest values (10,189 and 9480 µg/g DM, respectively). In flowers, total phenolics ranged from 4693 to 14,708 µg/g DM, with populations 3 and 4 emerging as the phenolic-richest (13,952 and 9899 µg/g DM, respectively). These results demonstrate substantial interpopulation variation in both the quantity and composition of phenolic and sulfur-containing compounds.

The pronounced chemical differences among populations reflect both flavonoid- and sulfur-based metabolic pathways that contribute to the species’ chemotypic diversity. Similar population-specific biochemical plasticity has been documented by Pavlović et al. [41] and Djurdjević et al. [42], who attributed such variability in *A. ursinum* to ecological heterogeneity and environmental stress gradients. The strong differences in allicin concentrations observed here further support the role of local environmental conditions in shaping sulfur-compound biosynthesis across natural populations

### 3.3. Principal Component Analysis (PCA)

PCA was applied to determine the phytochemical relationships between the studied populations of *A. ursinum*. PCA was performed using the combined concentrations of identified compounds in leaves and flowers across *A. ursinum* populations as independent variables in a two-dimensional score plot model. The first two principal components (PC1 and PC2) accounted for 52.8% of the dataset’s total variance.

**Figure 6:**
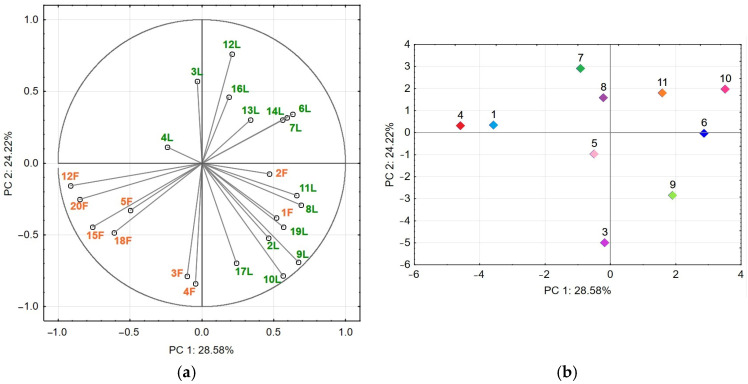
PCA represents eleven populations of *Allium ursinum* for the accumulation of bioactive compounds. Loading plots (**a**) for compound variables contributing to PC1 and PC2; Score plots for the testing populations (distinct populations in colors) (**b**). L—leaves (green); F—flowers (orange).

The results of the correlation matrices for the variables and the principal components PC1 and PC2 were presented in Figure 6. PC1 explained 28.58% of the total variance and was strongly positively correlated with compounds **6**, **7**, **8**, **9**, **11**, **14**, and **19** in leaves, as well as negatively correlated with compounds **12**, **15**, **18**, and **20** in flowers. PC2 accounted for 24.22% of the total variance in the dataset. It was significantly correlated with negative loadings for compounds **2**, **9**, **10**, and **17** in leaves and with positive loadings for compounds **3** and **4** in flowers. Additionally, it was positively correlated with compounds **3**, **12**, and **16** in leaves (Figure 6a).

The arrangement of *A. ursinum* populations 6, 10, and 11 can be explained by the significant contribution of variables that were positively correlated with PC1 and associated with the average amounts of leaf compounds within their range of variation (Figure 6b). On the other hand, the variables with high negative loadings on PC1 had the greatest impact on the clusters of populations 4 and 1, aligning with the negative PC1, and were associated with high levels of compound **1** in leaves and compound **12** in flowers, as well as moderate levels of compounds **15**, **18**, and **20** in flowers.

The positions of populations 3, 5, and 9 next to the negative PC2 were associated with high negative loadings of compounds **3** and **4** in flowers and compounds **2**, **9**, **10**, **17**, and **19** in leaves, indicating a range of variables from the mean to the highest values. Variables with high positive loadings on PC2 showed significant contributions to the position of populations 7 and 8. The position of population 7 coincided with the highest levels of compounds **3** and **12** in the leaves. Meanwhile, population 8 accumulated from the lowest to the average levels of the corresponding compounds within its range of variation.

Consequently, the PCA plot revealed the spatial distribution of populations based on their phytochemical profiles, and the principal components, PC1 and PC2, explained the primary directions of variation. The grouping patterns suggest that both plant part (leaf vs. flower) and population contribute significantly to phytochemical variability. Populations with similar compound profiles cluster together, while chemically distinct populations are positioned further apart.

### 3.4. Antioxidant Activity

**Figure 7:**
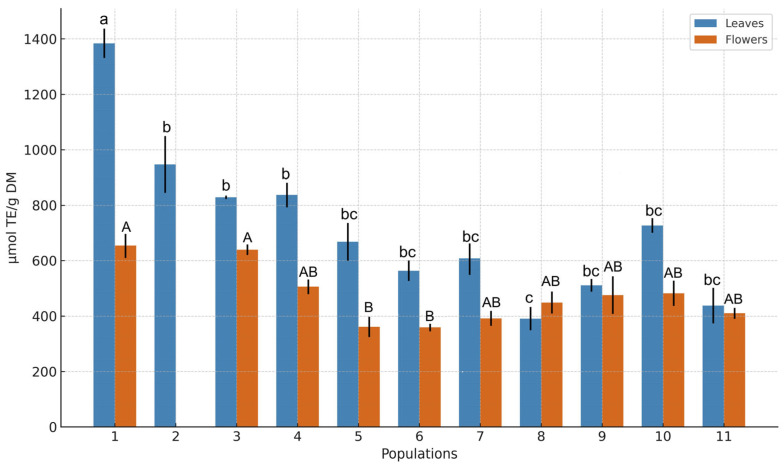
Comparison of mean Trolox equivalent antioxidant capacity values (µmol TE/g DM) in leaves and flowers of *Allium ursinum* across eleven populations. Columns (mean ± standard error) marked with different letters were significantly different between populations in leaves (lowercase) and flowers (uppercase) at *p* ≤ 0.05, according to the Kruskal–Wallis test. Flowers of population 2 were not analyzed due to a lack of raw material.

The results of the antioxidant activity of *A. ursinum* using the ABTS decolorization assay indicated significant differences between leaves and flowers, as well as between different populations (*p* < 0.05) (Figure 7).

The mean Trolox equivalent antioxidant capacity (TE) values for leaves were found to have priority over flowers (718.6 ± 282.8 vs. 473.0 ± 114.3 µmol TE/g DM, respectively). The relationship between radical-scavenging capacity and the values of the identified compounds was established using Pearson’s correlation analysis. Significant correlations were found between antioxidant activity and the content of specific kaempferol derivatives. A strong positive correlation (*p* < 0.05) was found between flower compounds **4**, **12**, **15**, and **18** and radical scavenging activities (*r* = 0.40, 0.46, 0.54, and 0.54, respectively). Meanwhile, the antioxidant capacity of leaf extracts showed a negative correlation with compounds **6**, **7**, **8**, and **11** (*r* = −0.52, −0.48, −0.48, and −0.49, respectively), the amounts of which ranged from low to medium. Populations 1, 3, and 4 demonstrated the highest antioxidant values in both plant parts, correlating with their dominant flavonol glycoside levels.

Furthermore, a moderate positive correlation (*r*^2^ = 0.42) was observed between antioxidant activity and total content of detected phenolics in flowers of *A. ursinum.* The correlation is reflected by an upward-sloping regression line with the equation TAC = 267.99 + 0.021 × TPC. The overall trend suggests that phenolic compounds are the main contributors to the antioxidant potential of floral tissues. However, no significant positive correlation was observed between total phenolics and TAC in leaves. This suggests that phenolics are not the main determinants of leaf antioxidant capacity or that other groups of compounds, such as carotenoids, or physiological factors, potentially moderate TAC. These differences indicate antioxidant mechanisms specific to plant organs, as flowers rely much more on phenolic compounds for antioxidant protection than leaves

Overall, the antioxidant capacity of *A. ursinum* extracts varied by plant part and population, along with differences in the phytochemical profile of individual compounds. Kaempferol derivatives appear to be the primary contributors to the antioxidant capacity of *A. ursinum* extracts.

### 3.5. Climate Conditions and Chemical Composition

**Figure 8:**
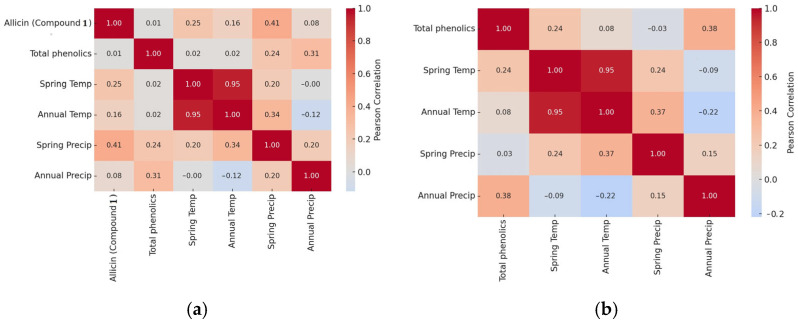
Pearson correlation heatmaps comparing associations among allicin, total phenolics, and climate variables in flowers (**a**) and total phenolics and climate variables in leaves (**b**) of *Allium ursinum*. Note: climate variables—spring temperature (Temp), annual temperature (Temp), spring precipitation (Precip), and annual precipitation (Precip). These findings suggest that moisture availability is a key limiting factor for the biosynthesis of secondary metabolites in *A. ursinum*. The findings suggest that moisture availability is a key limiting factor for the biosynthesis of secondary metabolites in *A. ursinum*.

The analysis of climate parameters at the sites of the studied populations revealed substantial differences in both average annual and spring precipitation (541.3–896.2 mm annually; 155.7–250.6 mm during February–May) and average air temperature (8.1–9.5 °C annually; 7.0–8.3 °C during February–May). These differences appear to correlate with variation in the accumulation of bioactive compounds. Populations in cooler and wetter environments (e.g., populations 3, 4, 7, 9, 10) tended to accumulate higher levels of total identified phenolics in leaves (e.g., 7280–10,189 µg/g DM), suggesting that spring precipitation and lower mean spring temperatures promote phenolic biosynthesis. Conversely, populations from drier and warmer areas (e.g., population 5—4637 µg/g DM) exhibited reduced phenolic levels in leaves. Flowers consistently showed higher phenolic concentrations than leaves, particularly in populations exposed to moderate precipitation and cooler conditions (e.g., populations 3, 7, 9). Meanwhile, allicin levels also varied significantly between populations, with higher levels typically accumulating at cooler, wetter sites (e.g., populations 3, 9, 10, 11). Since allicin is a sulfur-containing defense compound, its accumulation appears to be enhanced under conditions that increase plant stress. Correlation analyses confirmed that precipitation (both spring and annual totals) has a stronger influence on phenolic compound accumulation in flowers (Figure 8a). In contrast, annual precipitation in combination with spring temperature plays a greater role in leaves (Figure 8b). Furthermore, allicin shows a positive correlation with spring precipitation.

## 4. Discussion

The present study confirms and extends existing phytochemical knowledge of *A. ursinum* by identifying notable population-level and organ-specific differences in flavonol glycosides, lutein, and allicin, as well as the antioxidant activity of plant extracts. Overall, extracts of *A. ursinum* exhibited antioxidant capacity that varied by plant part and population, in parallel with differences in phenolic compound profiles.

Kaempferol glycosides were the dominant flavonol derivatives in both the leaves and flowers of *A. ursinum*, which is consistent with previous studies by different authors [28,37,38]. Among the identified compounds, a previously unreported quercetin glucuronide (compound **14**) was detected, thereby expanding the known phytochemical spectrum of *A. ursinum* and complementing previous profiling work [46,47].

Plant organs interacted significantly with kaempferol concentrations. Although both organs accumulated high levels of phenolics, flowers generally exhibited higher total concentrations of flavonol glycosides, while lutein and several unique kaempferol-acylated derivatives were more prevalent in leaves. The organ-specific accumulation pattern reflects tissue-specific metabolic regulation. It aligns with previous studies, which have shown higher carotenoid levels in leaves [39] and higher glycosylated flavonol levels in reproductive tissues [50,51]. Lutein was one of the dominant compounds out of the 11 carotenoids identified in wild garlic [39]. However, the presented results also revealed differences compared with some previous studies, which reported that kaempferol derivatives were predominant in leaves compared to other plant organs, such as flowers and bulbs [38,50], or seeds and stalks [37].

Although our results showed that the qualitative profile of kaempferol derivatives differed significantly between leaves and flowers, the total amount of identified compounds did not differ significantly. Kovačević et al. [52] and Kovarovič [50] also documented similar patterns, reporting that flowers often show higher total phenolic content due to the accumulation of flavonoid glycosides and sulfur-containing compounds. Our detection of allicin exclusively in flowers supports this pattern and echoes results from previous studies [26,40,51], which have emphasized allicin’s rapid degradation and sensitivity to extraction conditions. As shown by Stupar et al. [28], subcritical water extraction failed to detect allicin, which degraded into other sulfur species at high temperatures. It was shown that allicin can almost completely rearrange at 20 °C within 20 h to afford sulfur dioxide, and diallyl mono-, di-, and trisulfides, especially in organic solvents [32,53]. It should be noted that some discrepancies exist between studies regarding the organ-specific accumulation of phenolic compounds, flavonol glycosides, and sulfur metabolites in *A. ursinum*. While several studies, including the present one, report higher concentrations of flavonol glycosides in flowers [39,41], others have found greater amounts in leaves [35,42]. Such divergent findings may arise from multiple factors. First, the developmental stage at sampling plays a crucial role as the content of phenolic and sulfur compounds in *A. ursinum* is highly dynamic during its short phenological cycle. Early or late sampling during the spring season may yield significantly different compound profiles [38,54]. Second, differences in analytical methods, such as extraction procedures (e.g., solvent type, temperature, and extraction time), detection methods (HPLC conditions, detection wavelengths, etc.), and standards used for quantification can substantially affect measured concentrations and comparability across studies [33]. In addition, variability in microenvironmental conditions including soil nutrients, light intensity, canopy cover, and moisture, can differentially affect the metabolite accumulation in various tissues [26,55]. Furthermore, genetic variation among local ecotypes or genotypes may further exhibit distinct metabolic regulation patterns, as in wild-collected material, genetic diversity is substantial [42,56]. Finally, the instability of sulfur compounds such as allicin, post-harvest degradation, and extraction instability often result in inconsistent quantification between organs and studies [28,57]. Overall, these considerations highlight the need for standardized protocols and carefully controlled sampling designs when comparing phytochemical data across studies and plant organs.

Phenolic compounds, particularly glycosylated kaempferol derivatives, are the principal contributors to the antioxidant capacity in wild garlic [24,38,41]. Phytochemically profiled individual compounds showed correspondence to the radical-scavenging activity. Among individual compounds, kaempferol glycosides (compounds **4**, **12**, **15**, and **18**) showed the highest correlation with radical-scavenging capacity.

Differences in the antioxidant activity of polyphenolic compounds depend on their chemical structure, including the type and position of moieties. Structural characteristics such as the number of hydroxyl groups, degree of glycosylation, and presence of p-coumaroyl acylation play a key role in determining antioxidant potency [58]. Among flavonols, quercetin demonstrates the highest antioxidant activity, largely due to the catechol group in the B-ring and an extra hydroxyl group at the 3-position on the C-ring. However, kaempferol, which has only one hydroxyl group in the B-ring, does not exhibit significantly different antioxidant activity when compared to quercetin [59]. Most kaempferol derivatives are modified by O-glycosylation or acylation of hydroxyl groups, which typically reduces antioxidant activity [60]. In this way, highly glycosylated and heavily acylated derivatives, such as compounds **15** and **16**, typically show reduced radical-scavenging efficacy compared to free aglycones or lightly modified flavonols. On the other hand, glycosylation seems useful for enhancing water solubility, potentially improving bioavailability in vivo, and may partly compensate in biological systems, especially on the B ring [59].

The identified compounds **3**, **4**, **5**, **12**, **14**, and **15** are glycosylated at the 3- or 7-OH positions, thereby decreasing their radical-scavenging activity by masking the free hydroxyls essential for antioxidant effects. For example, compound 5 (kaempferol-3,7-di-O-glucopyranoside) showed a moderate level across populations, but likely contributed little to TAC due to full glycosylation. Compound **12** (kaempferol-3-O-rhamnopyranosyl-glucopyranosyl derivative) is a highly glycosylated form with limited antioxidant activity, but its high concentration (>400 µg/g DM) in some populations suggests quantitative relevance for TAC potential. Compounds **6**, **7**, **8**, **9**, **10**, **11**, and **13**, identified only in leaves, are acylated with phenolic acids (p-coumaroyl groups), which likely exhibit enhanced antioxidant activity due to the introduction of additional phenolic hydroxyls and restore some radical scavenging capacity despite glycosylation.

The complex forms of compounds **16**, **17**, **19**, and **20** (acetylated and coumaroylated kaempferol derivatives) retain substantial antioxidant potential, especially where p-coumaroyl moieties are present. Compound **18** (kaempferol-3-O-glucoside or astragalin) likely exhibits moderate antioxidant activity, which is primarily influenced by its structural characteristics. The aglycone core, kaempferol, provides basic antioxidant functionality, but glycosylation at the C3 position with a glucose moiety reduces antioxidant activity compared to free kaempferol [60].

O-glycosylated kaempferols, prevalent across leaf and/or flower profiles of all populations (e.g., compounds **3**, **5**, **12**), tend to show reduced radical scavenging activity due to the steric hindrance and masking of reactive hydroxyl groups. However, acylation with phenolic acids—especially p-coumaroyl moieties observed in compounds **6**, **13**, **16**, and **20**—may significantly enhance antioxidant potential by introducing additional reactive sites. This is evident in populations such as 7 and 10, where high levels of acylated compounds coincide with elevated TAC. Notably, the structure-activity relationship highlights the importance of acyl substitution patterns in modulating the antioxidant behavior of kaempferol derivatives.

While many studies of *Allium* species report a significant positive relationship between total phenolic content and TAC [61,62], several reports emphasize that this relationship is not universal and can be weak or absent under particular conditions [63,64]. The occurrence of weak/absence of correlation is mainly explained by assay and extraction methods, the high contribution of non-phenolic antioxidants present in garlic (especially thiosulfinates/allicin and ascorbate), and organ-specific metabolite profiles. These factors account for discrepancies across studies and are consistent with our observation that flowers and leaves exhibit different relationships between total phenolics and TPC.

Moreover, elevated antioxidant activity in populations may reflect not only phenolic content but also contributions from organosulfur compounds. Furdak et al. [65] confirmed synergistic effects between flavonols and sulfur constituents in *A. ursinum*, boosting both antioxidant and antiproliferative potential. In contrast to phenolic compounds, organosulfur compounds were found to have a negligible influence on the TAC of garlic and ramsons [66].

In contrast, sulfur compounds such as allicin contribute little to the overall antioxidant capacity, as confirmed in previous studies [53,54]. Allicin is almost exclusively responsible as a plant defense chemical for protecting plant tissues from herbivore and pathogen attacks [8,33,40]. As a consequence, the flower extract has a stronger inhibitory effect on some phytopathogenic fungi than the leaf extract [57], indicating its predominance in flowers, as confirmed in our study.

It is challenging to compare antioxidant activity measurements across studies of *A. ursinum* extracts due to variations in assay methods and units used to express total antioxidant capacity. Antioxidant capacity values obtained via the ABTS decolorization assay were significantly higher than those from the FRAP or DPPH assays [65]. Finally, the extraction method conditions can significantly influence antioxidant activity [67].

The present data confirm that *A. ursinum* in Lithuania occupies the full suite of mesic, nutrient-rich deciduous to mixed forests reported throughout Europe [68]. The inclusion of more xeric slope sites (populations 3 and 4) extends the known ecological amplitude, supporting reports of *A. ursinum* occurrence in drier yet still shaded microhabitats [69]. Furthermore, the mixture of *Alnus–Picea* and *Ulmus–Quercus* communities in Lithuania parallels both the alluvial floodplain forests of Eastern France [70] and Central European upland woods [7], indicating broad habitat plasticity within its core range.

While intra-specific chemical diversity has previously been recognized as an adaptive feature of *A. ursinum* [41,42], our data provides additional evidence that geographic and environmental heterogeneity, particularly climate and habitat characteristics, contribute to phytochemical variability. The research supports earlier findings that humid, shaded environments favor higher accumulation of secondary metabolites [18,38,71].

Previous studies show that allicin production is mainly independent of climatic conditions, while concentrations of kaempferol derivatives varied across a range of growing conditions [8]. However, other sources suggest that local habitat conditions may influence sulfur metabolism [26,57]. Allicin’s lability and rapid transformation mean that measured amounts are sensitive to both biological and handling factors. Nevertheless, sulfur metabolism is considered a defensive response to biotic/abiotic stress, suggesting that stressors related to the site microclimate increase with context [26,57,72]. Based on previous data and recent data, a conservative synthesis argues that phenolics show a clearer, positive relationship with moisture (and sometimes cooler spring temperatures) across locations. At the same time, allicin/sulfur profiles can vary widely and may only roughly correspond to average climate variables without more precise information on stress/behavior. PCA analyses further confirmed that climatic factors drive the clustering of phytochemical profiles among populations. This highlights the importance of integrating ecological and chemical data to understand adaptive responses of plant species. Although climatic parameters such as temperature and precipitation showed clear correlations with phenolic and sulfur compound variability, these factors alone may not fully explain the observed inter-population differences. Local environmental variables such as soil nutrient content, moisture, and light availability, as well as genetic diversity among natural populations, could also influence secondary metabolite profiles. Therefore, the present results should be interpreted as reflecting combined environmental and genetic influences rather than solely climatic effects. The study reflects results of a one-year study, so long-term monitoring is an important direction for future research, to complement the findings regarding the sustainable conservation and use of *A. ursinum*.

Overall, these findings highlight that both natural variability and environmentally induced changes in *A. ursinum* phytochemistry must be carefully considered for future applications in food, nutraceuticals, and potential therapeutic uses. In particular, understanding the role of climatic and habitat-specific factors will be essential for optimizing cultivation practices to obtain desired levels of bioactive compounds. For practical applications, cultivation or collection sites with moist, shaded forest environments and stable soil moisture appear most favorable for obtaining high-quality raw material rich in phenolics and sulfur compounds. Moreover, plants grow lushly in sufficiently moist, humus and nutrient-rich soils. In contrast, drier or warmer habitats may yield lower phenolic levels and reduced antioxidant potential. Such insights may support the selection of optimal growing regions and the development of site-adapted cultivation strategies for *A. ursinum* with enhanced phytochemical content.

## 5. Conclusions

This study reveals notable variation in the chemical composition and antioxidant activity of *Allium ursinum* populations across environmental conditions in Lithuania. The observed variability is closely linked to organ-specific metabolic pathways, population-level differentiation, and local climatic conditions. Populations exposed to higher spring moisture and moderate temperatures accumulated greater amounts of bioactive compounds. This suggests a strong influence of local climate on secondary metabolite synthesis in *A. ursinum*, but with organ-specific trends. The contents of phenolic compounds, particularly glycosylated kaempferol derivatives, correlated with antioxidant activity and were higher in populations growing under moist, shaded, and moderately cool conditions. Different relationships between phenolic compounds and antioxidant capacity were observed in leaves and flowers, indicating antioxidant mechanisms specific to plant organs, as flowers rely much more on phenolic compounds for antioxidant protection than leaves. These findings highlight the potential of environmental management in cultivation practices to optimize bioactive compound profiles in *A. ursinum*. Future studies integrating long-term ecological monitoring, genetic diversity assessment, and multi-location cultivation trials are recommended to support further sustainable utilization and commercial production of this valuable species. Research should include long-term population monitoring to assess annual metabolomic changes in the face of a changing climate and environmental conditions. A more detailed understanding of the contribution of genotypic diversity to phytochemical variability, taking into account the specific environmental factors that determine the successful survival of *A. ursinum*, is important for modulating the accumulation of bioactive compounds and their targeted application.

## Figures and Tables

**Table 1:** Description of sampling sites and habitats of the studied *Allium ursinum* populations in Lithuania.

Population No	Administrative Location	Latitude (°N) Longitude (°E)	Elevation (m.a.s.l.)	Habitat
1	Punia Pinewood, Alytus district	54.511944 24.016667	69	Mixed forest dominated by *Alnus glutinosa*, *Picea abies* and *Quercus robur*, with *Corylus avellana* undergrowth. Spring-fed lowland.
2	Prienai Pinewood, Prienai district	54.610055 23.942198	84	Mixed forest dominated by *P. abies*, *Tilia cordata* and *Q. robur*. Northern slope of a spring-fed stream.
3	Praveniškės Forest, Kaišiadorys district	54.938054 24.250098	71	Broad-leaved forest dominated by *Ulmus glabra* and *A. glutinosa*, with *C. avellana* undergrowth. A flat area by a stream
4	Ringiai Forest, Šilalė district	55.392127 22.240390	56–73	Broad-leaved forest dominated by *U. glabra* and *A. glutinosa*, with *C. avellana* undergrowth. Steep on the western slope.
5	Palnosai, Mažeikiai district	56.232485 22.578488	57–69	Mixed forest dominated by *A. incana*, *T. cordata,* and *P. abies*, with *C. avellana* undergrowth. Southern slope of the Venta River.
6	Cigonaliai, Kretinga district	55.943972 21.558562	56	Broad-leaved forest dominated by *Fraxinus excelsior*, *U. glabra*, and *A. incana*, with rich *C. avellana* undergrowth. A spring-fed lowland.
7	Šakiniai Forest, Klaipėda district	55.766167 21.535237	74	Mixed forest dominated by *P. abies* and *A. glutinosa*. Žvelsa river valley.
8	Biržai Forest, Biržai district	56.34343 24.87840	58	Mixed forest dominated by *P. abies*, *Betula pendula,* and *T. cordata*, with *C. avellana* undergrowth. A flat area by a stream.
9	Vilkaraistis Forest, Molėtai district	55.144265 25.343055	186	Mixed forest dominated by *A. incana* and *P. abies*, with *C. avellana* undergrowth. A spring-fed lowland.
10	Josvainiai Forest, Kėdainiai district	55.322160 23.820351	62	Mixed forest dominated by *U. glabra*, *B. pendula*, and *P. abies*, with *C. avellana* undergrowth. A flat area.
11	Padauguvos Forest, Kaunas district	55.078055 23.658602	65	Broad-leaved forest dominated by *U. glabra*, *P. abies,* and *Q. robur*, with *C. avellana* undergrowth. A flat area.

**Table 2:** Characterization of phenolic profiles of *Allium ursinum* using HPLC-MS.

No.	Retention Time (min)	Compound	m/z	UV_λmax_	Detected in a Plant Organ	References
1	7.682	Kaempferol 3-O-β-neohesperidoside-7-O-β-D-glucopyranoside	755, 593, 285	265, 346	F, L *	[8,38]
2	12.519	Kaempferol-hexosyl-acetyl-deoxyhexose-hexoside derivative	797, 635, 285	265, 346	F, L	[37]
3	19.179	Kaempferol-37-di-O-β-D-glucopyranoside	609, 447, 285	227, 265, 331	F	[37,38,46]
4	19.701	Kaempferol-deoxyhexose-hexoside-(p-coumaryl hexoside-hexoside)	1063, 593, 447, 285	226, 266, 315	L	[37,38]
5	20.54	Kaempferol-deoxyhexose-hexoside-(p-coumaryl hexoside-hexoside)	1063, 593, 447, 285	226, 266, 316	L	[37,38]
6	23.972	Kaempferol-hexose-(acetyl-deoxyhexose-(p-coumaryl hexosyl-hexoside)) derivative	1105, 635, 285	231, 266, 315	L	[37,38]
7	24.267	Kaempferol-hexose-(acetyl-deoxyhexose-(p-coumaryl hexosyl-hexoside)) derivative	1105, 635, 285	231, 266, 315	L	[37,38]
8	24.545	Kaempferol-hexose-(acetyl-deoxyhexose-(p-coumaryl hexosyl-hexoside)) derivative	1105, 635, 285	231, 266, 315	L	[37,38]
9	24.705	Kaempferol-hexose-(acetyl-deoxyhexose-(p-coumaryl hexosyl-hexoside)) derivative	1105, 635, 285	231, 266, 315	L	[37,38]
10	25.544	Kaempferol-3-O-rhamnopyranosyl-glucopyranosyl derivative	593, 285	265, 348	F	[37,38,46,47]
11	27.232	Kaempferol-(deoxyhexose-hexoside-(trans-p-coumaroyl)-hexoside) derivative	901, 593	230, 266, 315	L	[8,37,38,46]
12	28.294	Quercetin glucuronide ^st^	477	256, 351	L	Standard compound
13	30.233	Kaempferol-3-O-rhamnopyranosyl-acetyl glucopyranoside	635, 285	264, 348	F	[37,46]
14	30.265	Kaempferol-(acetyl-p-coumaryl-hexose)	635, 285	238, 265, 331	L	[37]
15	31.212	Acetyl-kaempferol-deoxyhexose-hexoside(p-coumaroyl)] hexoside derivative	943, 593, 447, 285	232, 266, 315	L	[37,38]
16	31.503	Astragalin ^st^	447, 285	264, 346	F	[38,46,47]
17	31.794	Acetyl-kaempferol-deoxyhexose-hexoside(p-coumaroyl)] hexoside derivative	943, 593, 447, 285	232, 266, 315	L	[37,38]
18	33.074	Kaempferol-(acetyl-p-coumaryl-hexose)	635, 285	265, 348	F	[37]

^st^ Chromatographic peak identification was carried out according to the analyte and reference compound retention time. * F—flowers; L—leaves.

**Table 3:** Mean content (µg/g DM) of allicin, lutein, and phenolic compounds (µg/g DM) in *Allium ursinum* leaves and flowers, their value range (min–max), and differences among *Allium ursinum* populations (*P*_1_) and between leaves and flowers (*P*).

No.	Compounds	Leaves			Flowers			*P* ^b^
		M ± SD	Min–Max	*P*_1_ ^a^	M ± SD	Min–Max	*P*_1_ ^a^	
1	Allicin	nd			1085.0 ± 330.3	622.3–1442.0	<0.001	
2	Lutein	535.0 ± 110.3	384.3–655.7	0.016	32.6 ± 8.3	25.6–46.4	0.002	<0.001
3	Kaempferol 3-O-β-neohesperidoside-7-O-β-D-glucopyranoside	910.7 ± 633.3	172.8–2550.5	<0.001	3446.8 ± 1236.6	1952.1–5949.0	<0.001	<0.001
4	Kaempferol-hexosyl-acetyl-deoxyhexose-hexoside derivative	2421.3 ± 1065.5	752.9–4045.0	<0.001	2303.1 ± 993.5	1304.8–4390.7	<0.001	0.571
5	Kaempferol-3,7-di-O-β-D-glucopyranoside	nd			149.5 ± 39.6	107.0–177.1	0.070	
6	Kaempferol-deoxyhexose-hexoside-(p-coumaryl hexoside-hexoside)	152.5 ± 73.3	64.8–280.9	<0.001	nd			
7	Kaempferol-deoxyhexose-hexoside-(p-coumaryl hexoside-hexoside)	453.9 ± 201.3	210.6–788.8	<0.001	nd			
8	Kaempferol-hexose-(acetyl-deoxyhexose-(p-coumaryl hexosyl-hexoside)) derivative	112.2 ± 40.0	56.8–180.5	<0.001	nd			
9	Kaempferol-hexose-(acetyl-deoxyhexose-(p-coumaryl hexosyl-hexoside)) derivative	144.7 ± 50.4	75.1–224.7	<0.001	nd			
10	Kaempferol-hexose-(acetyl-deoxyhexose-(p-coumaryl hexosyl-hexoside)) derivative	338.1 ± 112.2	195.6–514.8	<0.001	nd			
11	Kaempferol-hexose-(acetyl-deoxyhexose-(p-coumaryl hexosyl-hexoside)) derivative	445.5 ± 158.4	206.8–736.7	<0.001	nd			
12	Kaempferol-3-O-rhamnopyranosyl-glucopyranosyl derivative	191.1 ± 112.4	70.9–409.8	<0.001	1595.0 ± 797.9	586.1–2704.7	<0.001	<0.001
13	Kaempferol-(deoxyhexose-hexoside-(*trans*-p-coumaroyl)- hexoside) derivative	494.5 ± 270.2	160.9–1012.0	<0.001	nd			
14	Quercetin glucuronide	118.3 ± 99.7	8.2–358.4	<0.001	nd			
15	Kaempferol-3-O-rhamnopyranosyl acetylglucopyranoside	nd			473.7 ± 239.9	182.1–808.9	<0.001	
16.	Kaempferol-(acetyl-p-coumaryl-hexose)	110.4 ± 50.5	31.6–183.3	<0.001	nd			
17.	Acetyl-kaempferol-deoxyhexose-hexoside(p-coumaroyl)] hexoside derivative	719.7 ± 330.7	365.1–1439.3	<0.001	nd			
18	Astragalin	nd			514.7 ± 126.2	372.1–607.0	0.250	
19	Acetyl-kaempferol-deoxyhexose-hexoside(p-coumaroyl)] hexoside derivative	490.9 ± 194.1	280.1–773.2	<0.001	nd			
20	Kaempferol-(acetyl-p-coumaryl-hexose)	nd			177.3 ± 100.0	60.9–387.3	<0.001	

Values are means ± standard deviation. nd—not detected; ^a^ differences among populations by ANOVA; ^b^ differences between leaves and flowers according to *t*-test for dependent variables.

## Data Availability

The original contributions presented in this study are included in the article. Further inquiries can be directed to the corresponding author.
